# HTA supporting clinical practice: the case of surgical repair of thoracoabdominal aortic aneurysms

**DOI:** 10.1093/eurpub/ckac131.253

**Published:** 2022-10-25

**Authors:** GP Vigezzi, A Guddemi, D Bucci, S Colucci, D La Fauci, R Calsolaro, E Foglia, R Chiesa, L Bertoglio, A Odone

**Affiliations:** 1 Department of Public Health, University of Pavia, Pavia, Italy; 2 Ca’ della Paglia College, Ghislieri Foundation, Pavia, Italy; 3 School of Medicine, Vita-Salute San Raffaele University, Milan, Italy; 4 Administrative Direction, IRCCS San Raffaele Hospital, Milan, Italy; 5 HTA Commission, IRCCS San Raffaele Hospital, Milan, Italy; 6 LIUC Business School, LIUC Carlo Cattaneo University, Castellanza, Italy; 7 Division of Vascular Surgery, IRCCS San Raffaele Hospital, Vita-Salute San Raffaele University, Milan, Italy

## Abstract

**Background:**

Thoracoabdominal aortic aneurysms (TAAAs) are defined as those aortic aneurysms involving renovisceral arteries. They account for around 10% of aortic aneurysms, and their treatment is burdened by considerable mortality and morbidity. Open surgical repair has been practised as the standard of care since the 1950s. In 2001 endovascular repair was introduced to reduce treatment invasiveness, and the technology is still evolving. The potential benefits of endovascular repair over open surgery should be carefully weighed in a multidimensional framework.

**Methods:**

We applied the Health Technology Assessment (HTA) framework (EUnetHTA core model with 9 dimensions) to conduct a report comparing the two technologies. A multidisciplinary working group was established. We derived and pooled: i) secondary data derived from systematic literature reviews, and ii) original data from IRCCS San Raffaele Hospital, Milan, a national referral centre for TAAA (qualitative and economic data).

**Results:**

Endovascular repair resulted superior to the traditional open surgery in terms of efficacy and safety, as justified by the meta-analysis we performed. Despite the higher costs, a significant impact on budget and slightly lower cost-effectiveness, the endovascular protheses’ adoption could provide conspicuous benefits in terms of social and ethical dimensions without affecting long-term organisational and legal aspects.

**Conclusions:**

The multi-criteria decision analysis carried out from a hospital point of view shows that there is no significant difference (final score endovascular repair 0.68 vs open surgery 0.63) between the two procedures considering all the dimensions. Still, the endovascular repair is slightly superior to the open surgery in terms of safety, effectiveness, social, ethical, legal, and organisational impact. From the policy maker’s point of view, technologies with a score superior to 0.6 are equally valuable, and the final decision should be left to the clinicians.

**Key messages:**

• Further research is needed to compare endovascular prostheses and open surgery’s long-term population benefits, balancing clinical, economic, organisational and patient-related outcomes.

• HTA methodology offers substantial support to compare in-use technologies, informing clinicians’ and decision-makers’ choices to strengthen healthcare provision equity and preparedness.

